# Transgenerational effects from early developmental exposures to bisphenol A or 17α-ethinylestradiol in medaka, *Oryzias latipes*

**DOI:** 10.1038/srep09303

**Published:** 2015-03-20

**Authors:** Ramji K. Bhandari, Frederick S. vom Saal, Donald E. Tillitt

**Affiliations:** 1 U. S. Geological Survey, Columbia Environmental Research Center, Columbia, MO 65201, U.S.A; 2 Division of Biological Sciences, University of Missouri, Columbia, MO 65211

## Abstract

The transgenerational consequences of environmental contaminant exposures of aquatic vertebrates have the potential for broad ecological impacts, yet are largely uninvestigated. Bisphenol A (BPA) and 17α-ethinylestradiol (EE2) are two ubiquitous estrogenic chemicals present in aquatic environments throughout the United States and many other countries. Aquatic organisms, including fish, are exposed to varying concentrations of these chemicals at various stages of their life history. Here, we tested the ability of embryonic exposure to BPA or EE2 to cause adverse health outcomes at later life stages and transgenerational abnormalities in medaka fish. Exposures of F0 medaka to either BPA (100 μg/L) or EE2 (0.05 μg/L) during the first 7 days of embryonic development, when germ cells are differentiating, did not cause any apparent phenotypic abnormalities in F0 or F1 generations, but led to a significant reduction in the fertilization rate in offspring two generations later (F2) as well as a reduction of embryo survival in offspring three generations later (F3). Our present observations suggest that BPA or EE2 exposure during development induces transgenerational phenotypes of reproductive impairment and compromised embryonic survival in fish of subsequent generations. These adverse outcomes may have negative impacts on populations of fish inhabiting contaminated aquatic environments.

Environmental chemicals can induce a variety of adverse effects via disruption of normal endocrine function in eukaryotic organisms. These chemically-induced effects are not only observed as immediate organismal responses, but also as a variety of diseases in subsequent generations in organisms whose tissues were not directly exposed to the chemicals. The organism that develops abnormal health outcomes without direct chemical exposure, but rather because prior generations were exposed, is said to have a transgenerational phenotype. The transgenerational phenotype has been observed in many organisms, including humans and plants^1^. Since the observations by Anway *et al*^2^ that an agricultural fungicide, vinclozolin, can induce infertility and a variety of diseases in offspring three generations later, a variety of environmental toxicants (plastics, herbicides, fungicides, pesticides, dioxin, and hydrocarbons) and their mixtures have been shown to promote similar epigenetic transgenerational inheritance of adult-onset disease phenotypes in laboratory rats^3^,^4^. Epigenetic transgenerational inheritance of altered phenotypes, including those induced by contaminants, has also been shown in worms^5^, flies^6^, plants^7^, fish^8^,^9^,^10^, mice^11^,^12^ and humans^13^, although the mechanisms mediating these effects remain an active area of research.

Aquatic environments are the ultimate reservoirs for many environmental anthropogenic chemicals, including chemicals that mimic the functions of natural hormones. Fish and other aquatic organisms often have the greatest exposures to such chemicals during critical periods in development or even entire life cycles. Although immediate, deleterious outcomes of exposure to environmentally relevant concentration of endocrine disrupting chemicals (EDCs) have been reported for many aquatic species^14^, transgenerational effects resulting from chemical exposures are currently poorly understood. Declines of natural populations of fish and other aquatic species attributed to chemical contamination have largely been thought to be due to direct actions on the exposed organisms. However, the potential for transgenerational phenotypes to occur in aquatic organisms, consequently predisposing offspring of exposed lineages to a variety of diseases, remains an uncharacterized risk in natural populations^15^.

Bisphenol A (BPA) is a compound used primarily to manufacture polycarbonate plastics and epoxy resins, but is also an additive in other products^16^. Due to extensive use of these products in daily human life, the accumulation of BPA-containing waste in the environment has been a serious concern and a potential threat to the public and wildlife health^17^,^18^.

17α-ethinyl estradiol (EE2) is a component of combination oral contraceptives designed for women, of which approximately 16–68% of dose is excreted in the urine or feces^19^. A substantial amount of EE2 has been found in aquatic environments downstream of wastewater treatment plants. EE2 is strongly estrogenic, whereas BPA has weaker estrogenic activity via nuclear estrogen receptors. In contrast, the activity of BPA mediated by estrogen receptors associated with the cell membrane is equal to that of estradiol^20^. Both compounds are present in water at concentrations that are sufficient to affect development, osmoregulation, and reproduction in aquatic organisms^14^,^21^. Specifically, BPA occurs in the ng/L range in surface waters and up to the mg/L range in groundwater^14^, and once in sediment, does not readily degrade^22^. The concentration of EE2 in surface and ground water has been found in the high pg/L to low ng/L range (see review^14^). In the present study we examined whether these two chemicals of environmental and public health concern can induce transgenerational effects in aquatic organisms using medaka (*Oryzias latipes*) as a model species^23^,^24^,^25^,^26^,^27^ for studying germ cell mediated transgenerational effects. Medaka have advantages over other fish models, based on having all of these features: sequenced and annotated genome information, external fertilization and embryo development, daily spawning, availability of large numbers of eggs and sperm, short generation time (2 months) and easy, low-cost culture^25^,^28^. Moreover, medaka exhibits genetic sex determination, which occurs between days 5 and 7 after fertilization^29^, germ cell developmental events are well characterized^30^ and epigenetic reprogramming events in germ cells are believed to be complementary to the mouse.

## Methods

Additional details of methods used are available in the supplemental material section. All animal procedures were conducted in accordance with the procedures described by American Institute of Fishery Research Biologists (AIFRB), “Guidelines for Use of Fishes in Field Research”; and with all US Geological Survey CERC guidelines for the humane treatment of test organisms during culture and experimentation. Experimental protocols and the study plan was approved by USGS-CERC Institutional Animal Care and Use Committee.

### Animals and exposure

The Hd-rR strain of medaka (*Oryzias latipes*) maintained in the USGS Columbia Environmental Research Center (CERC) were used. Medaka eggs remain soft and less transparent during the unfertilized state and harden and become transparent after fertilization^31^. Due to transparency of fertilized egg, developmental stages can be examined under the light microscope. For the present study, fertilized eggs (F0) were collected from 6 to 10 breeding pairs, pooled, sorted, and assigned to 9 glass petri dishes. Each petri dish contained 50 fertilized eggs in 50 mL well water and each treatment group had three biological replicates. For EDC-treated fish, approximately 8 hours after fertilization (late blastula stage), well water was replaced by water containing the test chemicals BPA or EE2 at concentrations of 100 μg/L and 0.05 μg/L, respectively. The current exposure concentrations were above the levels currently measured in surface waters, but within expected environmental concentrations based on total estrogenicity assayed in surface waters and the potency of BPA and EE2^14^. Embryos were exposed for 7 days from that point spanning critical window of medaka sex determination^29^ which occurs between 5 and 7 days after fertilization. The control water and test chemical solutions were replaced daily until hatch. There were no further chemical exposures for these fish (F0) or any subsequent generations (F1, F2, F3, or F4). Newly hatched fry at 8–10 days postfertilization (dpf) were transferred to floating netted trays that were placed in 10 L aquaria with flowing water and aeration. At 30 dpf, all juveniles were transferred to aquaria and maintained. Culture of the three treatment lineages (control, EE2, and BPA) followed through production of F4 eggs and embryos.

### Chemical concentration measurement

Concentrations of EE2 in exposure waters were quantified by ELISA using a kit (Ecologiena EE2 ELISA kit, Tokiwa Chemical Industries, Tokyo, Japan) and manufacturer's instruction and concentrations of BPA by mass spectrometry according to previously published protocols^32^. To quantify the uptake of test chemicals into the medaka embryos, radioactive-labeled chemicals were used in a separate experiment, under identical conditions, with the same breeding pairs of fish used for the effect analysis portion of the study. Chemical concentrations in the exposure water and eggs for the uptake portion of the study were measured using liquid scintillation counting of ^3^H-EE2 and ^3^H-BPA [supplemental material].

### Mating, offspring production, and phenotype

**Figure 1:**
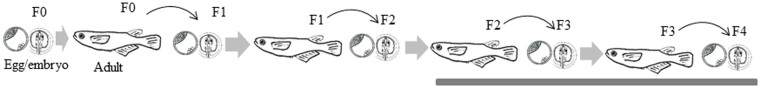
A schematic diagram showing multiple generations of medaka after early embryonic treatment with BPA (100 μg/L) or EE2 (0.05 μg/L). Transgenerational phenotypic traits examined are shown with a gray bar. The F2 adults had a transgenerational phenotypic trait of reduced fertilization capacity, whereas the F3 embryos that developed from fertilized eggs of F2 parents had reduced survival. Similar results were obtained in F3 adults and F4 embryos. All parts of the figure were drawn by Ramji Bhandari.

Water temperature was maintained at 26 ± 0.5°C throughout the study. All experimental medaka became sexually mature and spawned in an average of 120 dpf. A total of 6 adult pairs from the same treatment group were randomly chosen and mated to produce offspring for each subsequent generation (Fig. 1).

F0 fish were either unexposed (controls) or directly exposed to either test chemical, whereas F1 fish were exposed only as germ cells while in F0 gonads. Therefore, the first transgenerational phenotype was examined in offspring at the F2 generation. Eggs from six F2 breeding pairs of adults were collected over a 2-week period except for a few instances during which three out of six breeding pairs had spawned, counted, and sorted as fertilized or unfertilized. Fertilized eggs were placed in 50 mL petri dishes (n = 9) in well water with a daily change of 80% water until F3 embryos were hatched. The fecundity of the F2 adult breeding pairs, fertilization rate, and the survival rate of the F3 embryos were recorded. Similarly, eggs from F3 breeding pairs were collected and F4 embryos were produced. Fecundity was calculated as average (arithmetic mean) number of eggs produced by each breeding pair, fertilization rate as a percentage of fertilized eggs from total eggs collected in a day from each pair. The survival rate was calculated as the mean number of embryos that survived in the pool of fertilized embryos during embryonic development (before hatching). Phenotypic abnormalities were examined microscopically and gonadal changes histologically.

## Results

**Figure 2:**
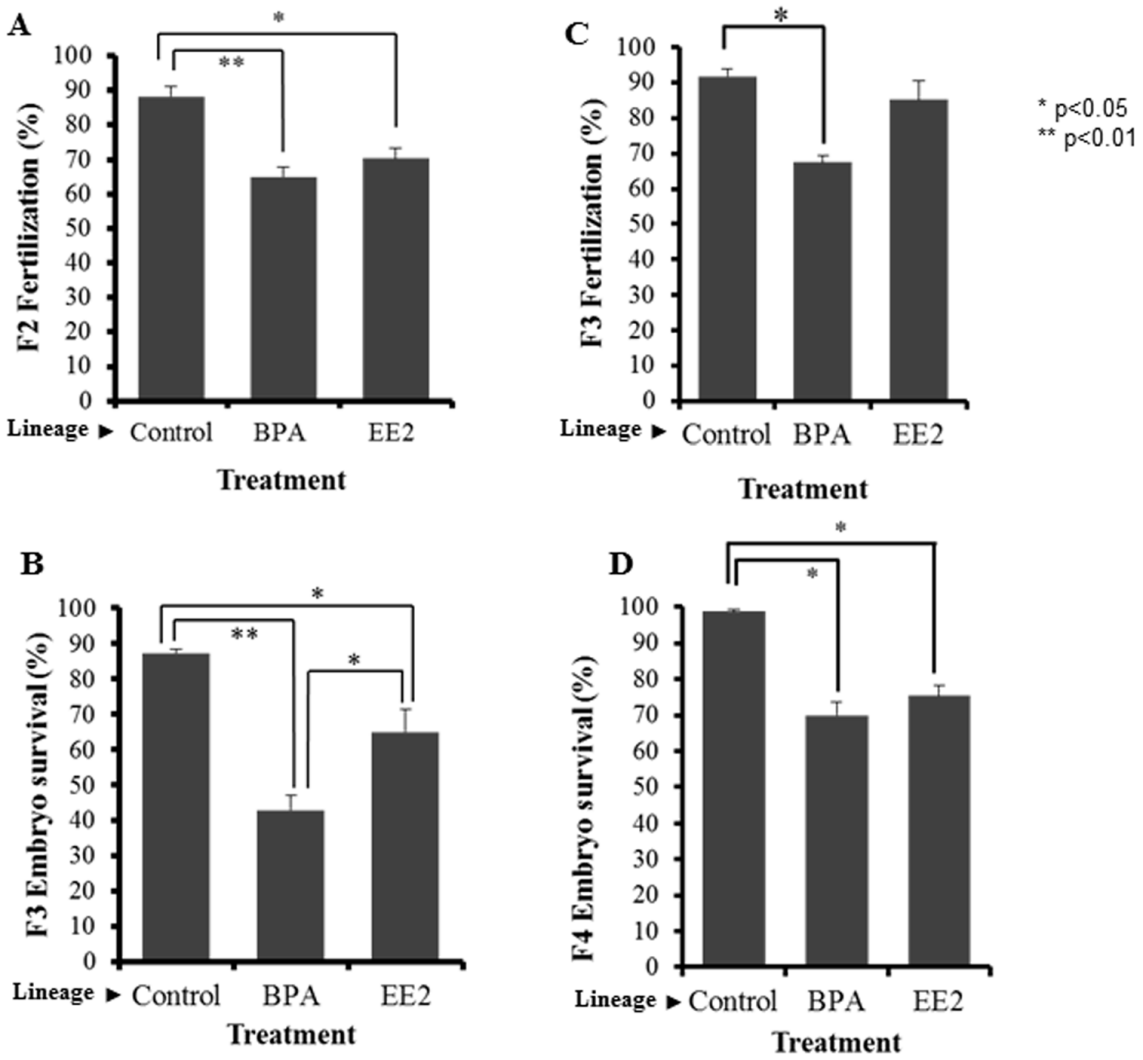
Transgenerational effects of BPA or EE2 exposures in fertilization capacity and survival of medaka. On the X axis, chemical names refer to corresponding lineages of F0 ancestors who were exposed during early development to exposure chemicals and never exposed after hatching. F2 fertilization capacity (A), F3 embryo survival (B), F3 fertilization capacity (C), and F4 embryo survival (D). BPA (100 μg/L) or EE2 (0.05 μg/L) exposures during embryonic development led to a significant reduction in fertilization capacity of breeding pairs and increase in embryo mortality in F2 through F4 generations. Statistical differences were calculated against same generation controls using a student's t-test; p values for these comparisons are as indicated (* p < 0.05, ** p < 0.01).

The measured water concentrations of BPA were 83.7 μg/L (16% less than nominal); while concentrations of EE2 were 0.061 μg/L (21% more than nominal water concentrations). The uptake of EE2 by embryos was 1.2 pg/mg egg in the first 24 hours and 4.0 pg EE2/mg egg in 7 days, whereas uptake of BPA by eggs was 29 pg/mg egg in 24 hours, 51 pg/mg egg in 3 days, and 178 pg/mg egg in 7 days (Supplemental table 1). All exposed F0 fertilized eggs survived and hatched. No phenotypic abnormalities were observed in F0 adults, with the exception of two fish with male to female sex reversal in EE2-treated group. The F0 adults, F1 embryos, F1 adults, and F2 embryos appeared to be normal in that they had no gross visible phenotypic changes. There was no significant difference in fertilization rate in F0 and F1 adults and in survival rate of F0, F1, and F2 embyros. The fertilization rate of eggs produced by the F2 generation was significantly reduced in BPA- and EE2-lineages (Fig. 2A). Embryo survival of F3 generation was significantly lower in both BPA- and EE2-lineages as compared to control treatments (Fig. 2B). The embryos that survived at F3 were maintained and F3 fertilization and F4 embryo survival were recorded. Compared to control, the fertilization rate of F3-BPA lineage was significantly reduced (p < 0.05) and F4 embryo survival decreased (p < 0.05) in both BPA- and EE2-linages (Fig. 1C&D). The fecundity of F1 EE2-lineage females was greater than in control- and BPA- lineage females (Fig. S2).

## Discussion

Environmentally induced transgenerational transmission of phenotypes at the organism or population level has been an area of recent intensive investigation^3^,^4^,^15^. In the present study we examined transgenerational effects of ubiquitous environmental estrogens (BPA and EE2) in medaka, a model fish. Our results indicate that exposure to a 100 μg/L concentration of BPA or a 2000-fold lower concentration (0.05 μg/L) of EE2 during 7 days of early embryonic development can produce transgenerational consequences, a reduced rate of fertilization and an increased incidence of embryo mortality. We also demonstrate that these transgenerational phenotypes appear in F2 generation adults and persist in F3 and F4 generations. In fathead minnows, EE2 exposure has been found to induce transgenerational effects on survival and fecundity, which consequently disrupted population dynamics^10^. Chemical compounds other than BPA and EE2 have been shown to cause transgenerational abnormalities in fish. Benzo-a-pyrene (BaP) exposure of adult male zebrafish resulted in transgenerational skeletal deformities in F1 and F2 generations^8^, whereas parental exposure to 1 μg/L BaP resulted in reduced egg production, reduced larval survival, and increased mortality of fathead minnows in the F2 generation^9^. Juvenile zebrafish exposed to dioxin (50 pg/mL in water for one hour at 3 and 7 weeks post fertilization) resulted in reduced fertilization success and egg release, and scoliosis-like skeletal abnormalities in the F2 generation^33^.

Toxicological studies that examined effects of environmental chemicals such as BPA and EE2 have identified several phenotypic abnormalities in fish and other species, with reproductive impairment among the reported phenotypic defects^14^. In a transgenerational study with rainbow trout, studies suggest that EE2 exposure in males caused diminished progeny survival in F1, but did not cause similar effects in F2 embryos^21^. However, no information is available on embryo survival at F3 and F4 generations. In the present study, the exposure did not produce any apparent reproductive abnormalities in the first two generations (F0 and F1), except for two F0 male to female instances of sex reversal in one of the three replicates of the EE2-treated group. The potential for EE2 to induce male to female sex reversal has previously been demonstrated^34^. Reduced rates of fertilization in adult F2 and F3 BPA-lineage fish and F2 EE2-lineage fish indicate that chemical exposures during development, which do not produce immediate effects in the exposed generation, can nevertheless negatively influence reproduction in later generations. We observed an approximate 30% reduction in F2 fertilization rate and a 20% reduction in F3 survival rates. If similar trends were observed in subsequent generations, a severe decline in overall population numbers might be expected by the F4 generation. Current findings from laboratory exposure studies are derived from highly controlled environments where the survival in test fish populations is greater than those in typical wild populations. In the environment, where survival of young fish is normally low, due to a multitude of natural confounding factors, such transgenerational effects on fish populations may indeed be more severe than the outcome reported here. Given that less than 200 pg BPA/mg egg and less than 5 pg EE2/mg egg induced such long-term effects in medaka reproduction, it seems important to revisit exposure situations and the risk posed to aquatic wildlife health.

Developmental origins of adult adverse health outcomes have been documented in many species, but little is known about how this developmentally programmed information is transmitted to subsequent generations with altered health conditions. Laboratory rodent studies indicate the involvement of epigenetic mechanisms such as DNA methylation and miRNAs in transgenerational inheritance of environmentally induced phenotypic traits^1^,^4^,^35^. Environmental chemical exposures, during the critical period of germ cell development, which coincides with the period of sex determination in many species, are believed to establish exposure-specific epigenetic marks in germ cells which either escape germ cell reprograming or are reestablished in each generation to cause phenotypic abnormalities in offspring several generations later^1^. In the present study, exposures were applied during embryonic development of medaka, within the critical period of medaka male sex determination, which is between day 5 and 7 after fertilization. Although the actual underpinning mechanisms are currently unknown, the transgenerational phenotypes caused by BPA or EE2 exposures in medaka may have been due to similar molecular changes in germ cells as has been observed with these chemicals in other taxa^11^,^12^,^36^,^37^. Future studies will examine molecular mechanisms underlying germline transmission of altered epigenome associated with these phenotypic changes using high-throughput genomic and epigenomic sequencing technologies.

The present study provides evidence for transgenerational reproductive abnormalities in medaka caused by developmental exposure to environmental estrogens, BPA and EE2. It will be important to understand if exposure of the developing fish embryo to other environmental estrogens can result in similar adverse outcomes, or whether chemicals such as BPA result in unique epigenomic profiles (mainly DNA methylation or histone marks or miRNA) due to acting as selective estrogen receptor modulators^38^. More importantly, it will be critical to determine if these transgenerational phenotypes occur in wild populations, under field exposure conditions. More detailed investigations into the mechanistic basis and physiological underpinnings of the observed transgenerational phenotypes will help answer these questions. Our findings call for studies to investigate presence of transgenerational phenotypes of adverse reproductive health outcomes in the natural population of fish and aquatic wildlife.

## Author Contributions

R.K.B., F.V.S. and D.E.T. designed the study, R.K.B. performed experiments, R.K.B. analyzed data, R.K.B., F.V.S. and D.E.T. prepared the manuscript. All authors reviewed the manuscript.

## Supplementary Material

Supplementary Information Supplemental Materials
