# Supplementary material for: Transgenerational effects from early developmental exposures to bisphenol A or 17α-ethinylestradiol in medaka, Oryzias latipes
Source: Sci Rep. 2015 Mar 20;5:9303. doi: 10.1038/srep09303 (PMC4366817; doi:10.1038/srep09303)
Supplement: Supplementary Information — Supplemental Materials [file srep09303-s1.pdf]

SUPPLEMENTARY MATERIALS

Manuscript title: Transgenerational effects from early developmental exposures to bisphenol A or 17α-ethinylestradiol in medaka, *Oryzias latipes*

Ramji K. Bhandari[‡†*], Frederick S. vom Saal[†], Donald E. Tillitt[‡]

[‡]USGS Columbia Environmental Research Center, Columbia, MO 65201, U.S.A.

[†]Division of Biological Sciences, University of Missouri, Columbia, MO 65211

Materials and Methods

1) ^{3}H-EE2 measurement in eggs and dosing solution

EE2 dosing solution was prepared with filtered well water at a concentration of 0.05 µg EE2/L water. EE2 concentrations in the dosing solution were quantified by ELISA using a kit (Ecologiena EE2 ELISA kit, Tokiwa Chemical Industries, Tokyo, Japan) and manufacturer's instruction. EE2 uptake by eggs was measured using ^{3}H-EE2 (American Radiolabeled Chemicals, Inc). The dosing solution was prepared at a concentration of 0.05 µg EE2/L filtered well water. Medaka fertilized eggs were collected and placed in 50 mL glass petri dishes and 50 embryos were treated with the dosing solution of ^{3}H-EE2. Concentration of ^{3}H-EE2 was measured in initial dosing solution, dosing solution at the time of dosing, at the time of 70% replacement in 24 hours. Fertilized eggs or embryos were dosed for 7 days. Uptake was measured at day 0, 1, and 7 of exposure in a scintillation counter (Beckman, Germany).

2) ^{3}H-BPA measurement in eggs and dosing solution

BPA dosing solution was kindly measured Dr. Julia Taylor by mass spectrometry according to Taylor *et al* (2011) and scintillation counting. ^{3}H-BPA (> 99% pure; Sigma-Aldrich) was used at a concentration of 10 µg/L. Total of 20 fertilized medaka eggs and 10 mL glass beakers were used. Uptake was measured at day 0, 1, 3, and 7 of exposure. All other procedure of dosing and scintillation counting was similar to that used for ^{3}H-EE2 measurement.

REFERENCE

Taylor, J.A., Vom Saal, F.S., Welshons, W.V., Drury, B., Rottinghaus, G., Hunt, P.A., Toutain, P.L., Laffont, C.M. & VandeVoort, C.A. 2011 Similarity of bisphenol A pharmacokinetics in rhesus monkeys and mice: relevance for human exposure. Environmental health perspectives 119, 422-430.

Supplemental Table 1. Concentration of the test chemicals and uptake of test chemicals by eggs in different time points. The EE2 uptake was measured only at two time points (24 hr and 7 days).

	Concentration (ug/L)	
Test chemical	Nominal	Measured
BPA	100	83.70
EE2	0.05	0.06

	Uptake by medaka eggs (pg/mg egg)		
	24 hr	3 days	7 days
^{3}H-BPA	28.6	51	178
^{3}H-EE2	1.2	-	4.0

Supplementary figures

Fig. S1

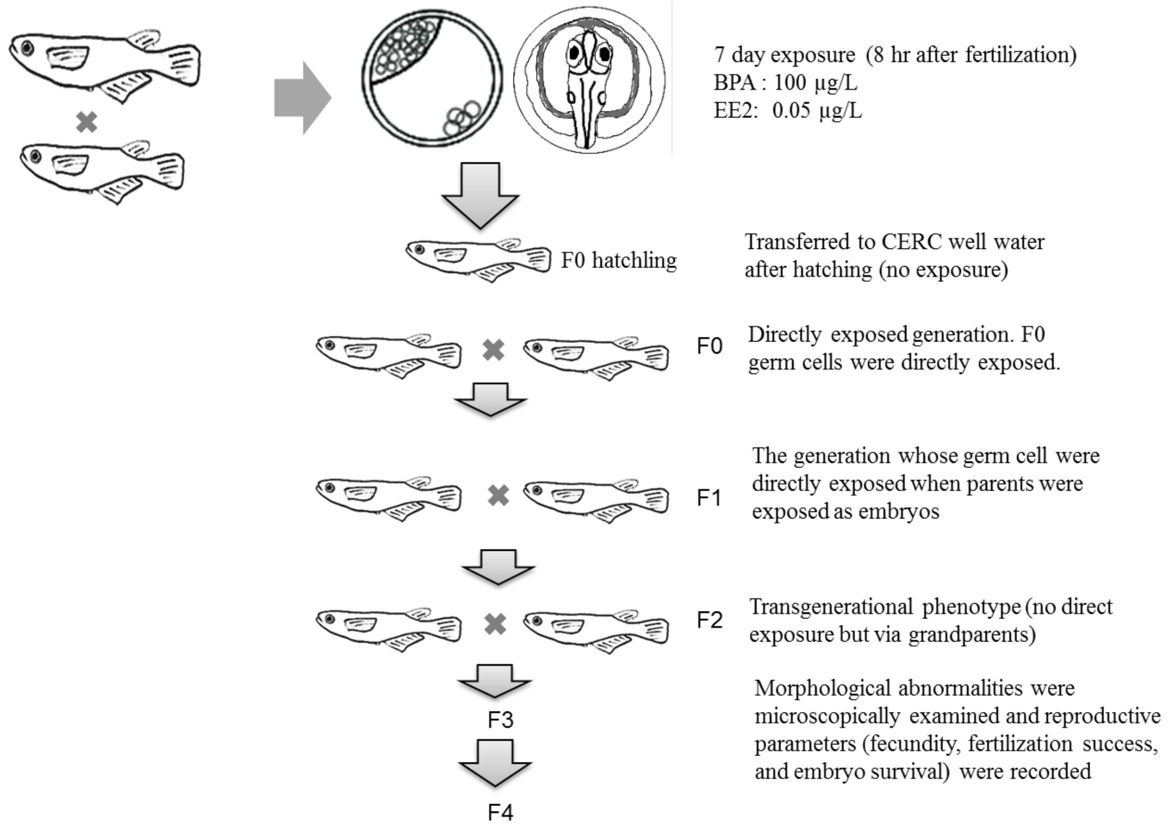

Fig S1. The experimental design with treatment and mating strategies. Fertilized eggs gave rise to F0 adults. F0 were directly exposed for 8 days during embryo development, thus F1 were exposed as germ cells when F0 were developing. Only the F2 generation fish which were not directly exposed were considered as individuals with transgenerational phenotypic traits. In each generation, sibling mating was performed to reflect a natural situation. No parent-of-origin studies were designed for this project.

Fig. S2

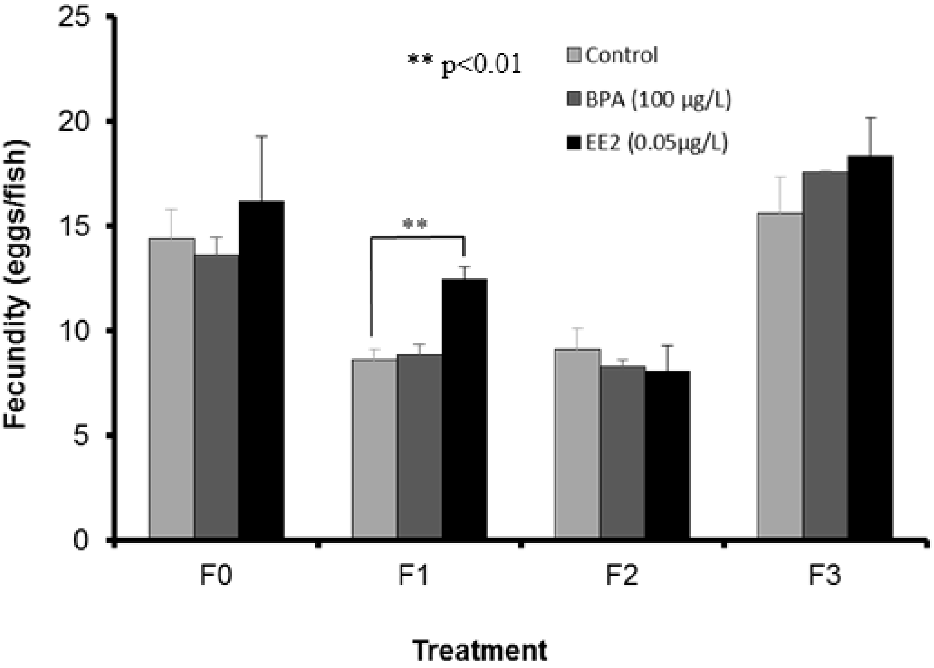

Fig. S2. Transgenerational effects of BPA and EE2 treatments on egg production of medaka breeding pairs. BPA (100 µg/L) and EE2 (0.05 µg/L) treatments during embryonic did not cause significant differences in egg production of the breeding pairs, except for the pairs in F1 EE2-treated lineage. Statistical differences were calculated against same generation control values using student's t-test at p values as indicated (** $P<0.01$).
